# Eat, play, live: a randomized controlled trial within a natural experiment examining the role of nutrition policy and capacity building in improving food environments in recreation and sport facilities

**DOI:** 10.1186/s12966-019-0811-8

**Published:** 2019-06-25

**Authors:** Dana Lee Olstad, Kim D. Raine, Rachel J. L. Prowse, Dona Tomlin, Sara F. Kirk, Jessie-Lee D. McIsaac, Louise C. Mâsse, M. Susan Caswell, Rhona M. Hanning, Todd Milford, Patti-Jean Naylor

**Affiliations:** 1 Department of Community Health Sciences, Cumming School of Medicine, University of Calgary, 3280 Hospital Drive NW, Calgary, AB T2N 4Z6 Canada; 2 School of Public Health, University of Alberta, 11405 87 Avenue NW, Edmonton, AB T6G 1C9 Canada; 3 School of Exercise Science, Physical and Health Education, University of Victoria, PO Box 3015 Stn CSC, Victoria, BC V8W 3P1 Canada; 4 Healthy Populations Institute, Dalhousie University, Stairs House, PO Box 15000, 6230, South Street, Halifax, NS B3H 4R2 Canada; 5 Faculty of Education and Department of Child and Youth Study, Mount Saint Vincent University, 166 Bedford Hwy, Halifax, NS B3M 2J6 Canada; 6 BC Children’s Hospital Research Institute, School of Population and Public Health, University of British Columbia, 4480 Oak Street, Vancouver, BC V6H 3V4 Canada; 7 School of Public Health and Health Systems, University of Waterloo, 200 University Ave West, Waterloo, ON N2L 3G1 Canada; 8 Department of Curriculum and Instruction, Faculty of Education, University of Victoria, PO Box 1700, STN CSC, Victoria, BC V8W 2YW Canada

**Keywords:** Children, Food environments, Recreation and sport facilities, Policy, Capacity building, Public health

## Abstract

**Background:**

Recreation and sport facilities often have unhealthy food environments that may promote unhealthy dietary patterns among children. In response, some Canadian provinces have released voluntary nutrition guidelines for recreation and sport facilities, however implementation has been limited. Organizational capacity building may overcome barriers to implementing guidelines. Eat, Play, Live was a randomized controlled trial embedded within a natural experiment that tested the impact of an 18 month capacity building intervention (CBI) in enhancing implementation of provincial nutrition guidelines, and whether nutrition guidelines were associated with positive changes. Primary outcomes were facility capacity, policy development and food environment quality.

**Methods:**

Recreation and sport facilities in three guideline provinces were randomized into a guideline + CBI (GL + CBI; *n* = 17) or a guideline only comparison condition (GL-ONLY; *n* = 15). Facilities in a province without guidelines constituted a second comparison condition (NO-GL; n = 17). Facility capacity, policy development, and food environment quality (vending and concession) were measured and compared at baseline and follow-up across conditions using repeated measures ANOVA and Chi-square statistics. Healthfulness of vending and concession items was rated as Do Not Sell (least nutritious), Sell Sometimes or Sell Most (most nutritious).

**Results:**

There were significant time by condition effects, with significant increases in facility capacity (mean ± SD: 30.8 ± 15.6% to 62.3 ± 22.0%; *p* <  0.01), nutrition policy development (17.6% developed new policies; *p* = 0.049), overall quality of the concession food environment (14.7 ± 8.4 to 17.5 ± 7.2; *p* <  0.001), and in the proportion of Sell Most (3.7 ± 4.4% to 11.0 ± 9.0%; *p* = 0.002) and Sell Sometimes vending snacks (22.4 ± 14.4% to 43.8 ± 15.8%; p <  0.001) in GL + CBI facilities, with a significant decline in Do Not Sell vending snacks (74.0 ± 16.6% to 45.2 ± 20.1%; p <  0.001).

**Conclusions:**

Significant improvements in facility capacity, policy development and food environment quality occurred in recreation and sport facilities that were exposed to nutrition guidelines and participated in a CBI. Outcomes did not improve in facilities that were only passively or not at all exposed to guidelines. Ongoing capacity building may enhance implementation of voluntary nutrition guidelines, however food environments remained overwhelmingly unhealthy, suggesting additional scope to enhance implementation.

**Trials registration:**

Clinical trials registration (retrospectively registered): ISRCTN14669997 Jul 3, 2018.

**Electronic supplementary material:**

The online version of this article (10.1186/s12966-019-0811-8) contains supplementary material, which is available to authorized users.

## Introduction

Sports and unhealthy food and drink - the former promotes health, while the latter can undermine it; yet in many cases the two are closely associated. This is no less the case in Canada, where publicly funded recreation and sport facilities (community facilities with physical activity-related infrastructure and programming) often have unhealthy food environments, despite their mandate to promote health [1–4]. The preponderance of unhealthy food environments in these facilities is a concern because a majority of users are children who, by virtue of their immaturity, may be particularly vulnerable to negative environmental exposures [5, 6]. Indeed, whereas in adults positive health behaviours tend to cluster [7, 8], children involved in sport actually consume more sugar sweetened beverages, fast food and energy relative to children who are less active [9]. Moreover, compared to their mothers, children are more likely to consume unhealthy foods during periods of physical activity [10]. These differences might plausibly relate to the unhealthy nature of the food environment in many community recreation and sports settings and suggest a need for remedial action.

The socioecological model can provide a foundation for understanding potential leverage points to improve children’s dietary intakes [11]. Policy constitutes the outermost sphere of the socioecological model because it establishes the default conditions for all other levels; it shapes organizational and community environments which in turn influence individuals’ personal capabilities and resources. Given its pervasive influence, public policy governing food availability, accessibility and promotion has therefore emerged as a top priority to improve children’s food environments worldwide [12]. However, while action to develop and implement nutrition policies has occurred in schools, other public settings such as recreation and sports facilities have received limited attention. Notably, three Canadian provinces (equivalent to states) stand out as among few jurisdictions worldwide that have taken action by releasing nutrition guidelines, albeit of a voluntary nature, for recreation and sport facilities [13–15]. Although voluntary guidelines may lack the potency of mandatory policies, we have previously shown that recreation and sport facilities located in provinces with nutrition guidelines had healthier food environments than facilities in a province without nutrition guidelines (unpublished observations). Nevertheless, food environments remained overwhelmingly unhealthy in all cases, suggesting that voluntary nutrition guidelines alone are not sufficient to create truly healthy food environments, and that additional supports are required to overcome barriers to their implementation [2, 4, 16, 17].

Organizational capacity building within recreation and sport facilities offers potential to overcome barriers to implementing provincial nutrition guidelines (e.g. perceived lower profitability of healthier items, limited knowledge and skills to implement nutrition guidelines, minimal stakeholder buy-in and cooperation [1, 2, 4, 18, 19]). Previous capacity building interventions (CBI) have supported organizations in successfully addressing health-related issues by enhancing local commitment, knowledge, skills, leadership, structures and systems [20]. Because they rely largely on existing resources and supports, CBIs do not require new, costly infrastructure or bureaucracies, thereby enhancing their feasibility. We have previously shown that when provincial nutrition guidelines were in place, 8 months of capacity building activities focussed on improving problem recognition, engaging key stakeholders, and providing training, resources, and supports were associated with positive change in recreation and sport facility capacity, policy development and food environments [2]. Given promising results from this quasi-experimental controlled study (i.e. facilities self-selected to participate in a CBI and were compared to facilities that volunteered to act as comparison facilities), we perceived a need to study the impact of a more comprehensive, longer CBI scaled up to a national level, and to use a stronger randomized, controlled design capable of supporting causal inference.

**Fig. 1:**
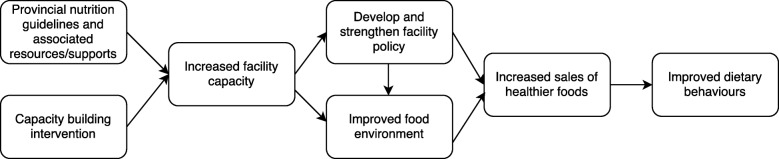
Logic model

Thus, the purpose of the Eat, Play, Live (EPL) trial was to provide longitudinal evidence of the impact of an 18 month CBI in enhancing implementation of provincial nutrition guidelines across multiple provincial contexts. Primary outcomes were recreation and sport facility capacity, policy development and food environments. Figure 1 presents the hypothesized pathway from intervention to outcomes. We also examined whether nutrition guidelines were associated with positive change in these outcomes over time. Our overall aim was to test whether a relatively small investment in building capacity could protect government’s much larger investment in developing and disseminating voluntary nutrition guidelines, and the level of implementation that could realistically be achieved within a voluntary policy framework.

## Methods

### Study design and context

**Fig. 2:**
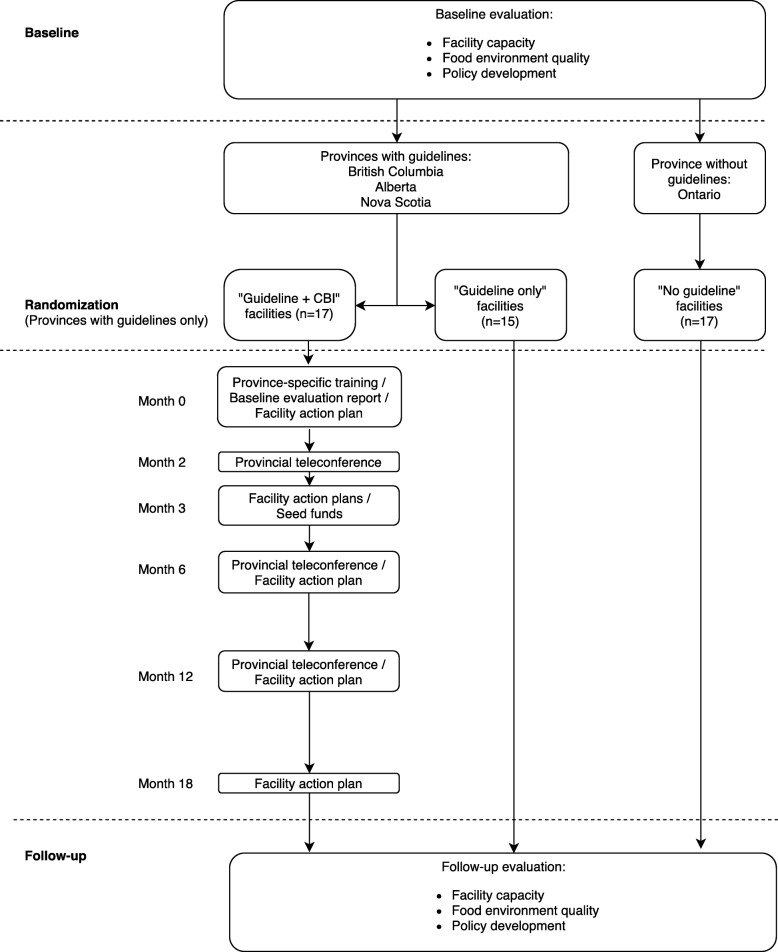
Study flow chart

EPL was a RCT embedded within a natural experiment that leveraged differences in nutrition policy/guidelines for recreation and sport facilities among Canadian provinces. Policies are defined as a “relatively stable, purposive course of action followed by an actor or set of actors in dealing with a problem or matter of concern” [21] and can include both formal and informal rules, laws and regulations [22]. The terms policy and guidelines are therefore used interchangeably throughout this paper. Participating facilities in the three Canadian provinces with nutrition guidelines for recreation and sport facilities at the time of the study (British Columbia = BC, Alberta = AB, Nova Scotia = NS) [13–15] were randomized to an 18 month CBI or a guideline only comparison condition (i.e. no CBI). Facilities in a province that did not have nutrition guidelines for recreation and sport facilities (Ontario = ON) constituted a second comparison condition that was not randomized (Fig. 2). ON was selected as a no guideline comparison province as it provided the closest match in terms of population size, urban/rural mix, economic activity, language and culture.

The study received ethical approval from the Health Research Ethics Boards at the University of Victoria, the University of British Columbia, the University of Alberta, the University of Waterloo and Dalhousie University. Managers of recreation facilities agreed to their facilities’ participation. This manuscript adheres to CONSORT (Additional file 1) and TIDieR (Additional file 2) reporting standards.

### Recruitment

**Fig. 3:**
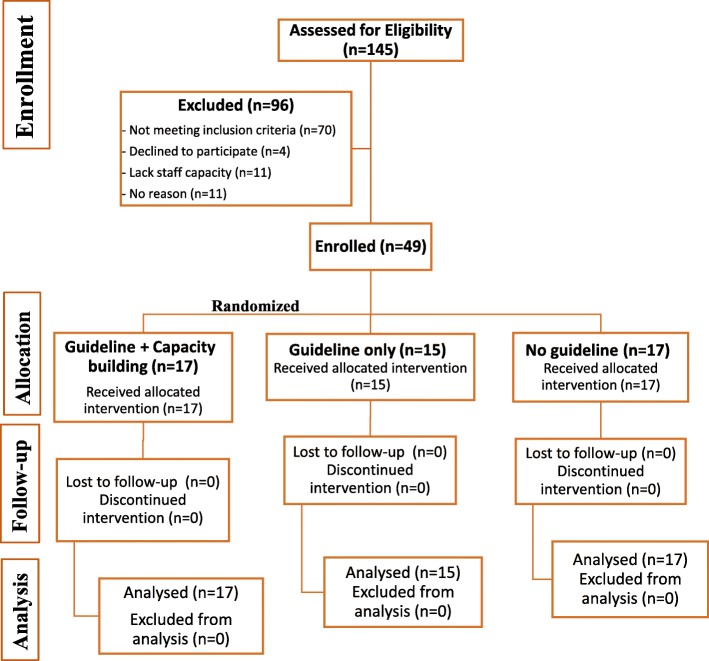
CONSORT flow diagram

Recreation and sport facilities were recruited by linking with each province’s respective Recreation and Parks Association (or a similar organization) to initiate website postings, email their members and provide presentations at their annual meetings. Publicly funded municipal recreation and sport facilities were eligible to participate if they: 1) Had not been involved in an intervention to improve their food environment since 2010; 2) Offered food/beverages through vending machines and/or a concession (analogous to cafeterias, canteens and kiosks); 3) Had the ability to change their food environment; and 4) Offered recreational programming, preferably to children. Facilities located in close proximity to the coordinating Universities were preferred. A total of 286 facilities indicated an interest in participating, of which 145 returned our phone calls/emails. Of the 145 facilities contacted, 75 facilities were eligible and 49 facilities enrolled (65%) (Fig. 3). Reasons for non-participation were: 11 lacked staff capacity, two were not interested in participating in research, one was unwilling to potentially be allocated to a comparison condition, one was afraid of losing revenue and 11 provided no reason.

### Allocation to condition

Three parallel groups were included (Fig. 2). Following collection of baseline data, an independent researcher used a 1:1 allocation ratio to randomize the 32 facilities located in guideline provinces into a guideline + CBI (*n* = 17) or a guideline only comparison condition (*n* = 15). A computer-generated randomization sequence stratified by province, food service types (i.e. vending only, concession only, mixed vending and concession), and participation in pre-2010 interventions was used. One extra facility was allocated to the guideline + CBI group in AB and NS, as there was an uneven number of facilities in these provinces. Allocation concealment was ensured via secure storage of the randomization sequence separately from the facility database, and was only accessible to the researcher who performed the randomization. All 17 facilities in the province of ON constituted the no guideline comparison group. The unit of randomization and analysis was individual recreation and sport facilities. Facilities could not be blinded to group assignment.

1. The **no guideline comparison group** (NO-GL) consisted of facilities in ON, a province that did not have nutrition guidelines for recreation and sport facilities. These facilities had the potential to be passively exposed to all publicly available nutrition guidelines and resources (e.g. online toolkits), however a manipulation check confirmed that prior to the study none were aware of guidelines in other provinces, nor did they investigate them during the course of the study. These facilities were not randomized but were assigned ‘by nature’ to condition, and thus did not self-select their condition.
2. The **guideline only comparison group** (GL-ONLY) consisted of facilities in BC, AB and NS that were passively exposed to their respective provincial nutrition guidelines and associated resources, along with all other publicly available guidelines and resources. These facilities were randomized to condition.
3. The **guideline + capacity building intervention group** (GL + CBI) consisted of facilities in BC, AB and NS. These facilities were subject to the same provincial context and guideline exposures as the guideline only group, and received an actively delivered 18 month CBI. These facilities were randomized to condition.

### Intervention

#### Provincial nutrition guidelines

Voluntary nutrition guidelines for recreation and sport facilities were introduced at different times in the three Canadian provinces, with NS in early [15], AB at mid [14] and BC [13] at a later stage of guideline implementation at the time of the study. All three provincial guidelines provide a basis to classify the healthfulness of foods using nutrient profiling schemes, along with guidance as to how facilities can support healthy eating by increasing the availability, accessibility and promotion of healthier foods. There is moderate to good agreement between nutrition profiling schemes in BC, AB and NS [23], although some aspects of some schemes were recently modified by their respective governments. Facilities in each province could access a variety of province-specific supports to facilitate guideline implementation, which primarily consisted of online toolkits and implementation guides.

#### Capacity building intervention model, process and components

The overall CBI model, previously developed and refined in BC [2, 24], was modeled after the linking system approach [25, 26]. The CBI was designed to link researchers (the ‘resource group’) with recreation facility staff (the ‘user group’). A Provincial Coordinator in each guideline province served as the ‘linking agent/knowledge broker’ and provided training and technical support as facilities created localized facility action plans. Provincial Coordinators included two Registered Dietitians/PhD Candidates and one MSc-level Research Associate. All three were trained in health promotion and nutrition and had conducted previous research in recreation and sport facilities. Provincial Coordinators also linked facilities with resources that were available to support their work (e.g. online toolkits, published evaluations, public health dietitians), and provided feedback on their progress. In this way, the Provincial Coordinator facilitated the translation of knowledge into action [27]. It was envisioned that lessons learned by the ‘user group’ would influence further actions within and across sites because of opportunities for cross-site sharing embedded within the CBI.

The specific elements of the CBI were informed by the implementation science literature [25, 26, 28] and our previous research in recreation and sport facilities [1, 2, 4, 24, 29–31] (Table 1). It represents the combination of elements that proved to be both acceptable to stakeholders and crucial to intervention effectiveness. The purpose of the CBI was to build local stakeholder capacity for implementing provincial nutrition guidelines through a series of five suggested steps executed over the course of 18 months: 1) Receive a half-day of province-specific training from Provincial Coordinators (facility managers or other designates were trained in an in-person group setting immediately after randomization); 2) Form a local planning team; 3) Review baseline evaluation report (i.e. facility capacity and food environment quality); 4) Create an individualized facility action plan; and 5) Implement, monitor and adjust the action plan. The implementation process (i.e. steps 2–5) was to unfold organically within individual facilities with limited involvement from researchers. Researchers did not prescribe certain changes; instead, facilities created, revised and executed their own contextualized action plans consistent with their priorities. Provincial Coordinators provided on-demand support and maintained regular contact with facilities (email, phone, in-person) at least monthly. Seed funds in the amount of $1000 CAD were also provided 3 months after training to each facility to invest in their food change activities, and all study-associated travel was reimbursed. Provincial (2, 6, 12  months) teleconferences supported cross-site sharing. At the end of the study, NO-GL and GL-ONLY facilities were offered a CBI consisting of the initial CBI training session, access to an online toolkit and 6 months of on-demand support. Many of the CBI resources have now been made publicly available at: https://stayactiveeathealthy.ca/website-resources/.

**Table 1:** Capacity building intervention (CBI) elements for the Eat, Play, Live trial

CBI resources and supports	Frequency and timing	Description
Province-specific training	Once, 0 months	Half-day, in-person group training covering the following: • Five-step change process
		• Operational areas for policy and program changes in food environments
		• Practical examples of food environment changes for each operational area
		• Provincial nutrition guidelines
		• Resources to support food environment and policy change (see below)
		• CBI protocol and timeline
Baseline evaluation report	Once, 0 months	All facilities received a report summarizing data collected at baseline in their facility on facility capacity and food environment quality
Seed funds	Once, 3 months	$1000 CAD per facility
Online toolkit	Unlimited access	BC Recreation and Parks Association website (stayactiveeathealthy.ca) provides information on healthy food in recreation. Includes searchable database of resources and provincial pages with local resources
Online resources	Unlimited access	Local resource hubs to support environment and policy change were provided in BC (https://bnfl.healthlinkbc.ca/), AB (healthyeatingstartshere.ca; apccprecproject.com) and NS (https://thrive.novascotia.ca/, https://thrive.novascotia.ca/resources)
Provincial Coordinator support	Monthly follow-up and as needed	On-demand individual training, resources, and support (e.g. assistance with nutrition analysis, product sourcing, connecting with community resources including public health dietitians) via email, telephone and in-person
Provincial teleconferences	2, 6, 12 months	60 min intra-provincial cross-facility meetings; facilities described current and planned activities, challenges and facilitators
Facility action plans	0, 3, 6, 12, 18 months	CBI facilities developed written implementation plans to achieve their goals
Electronic information provision	Every 1–2 months	Periodic group emails from Provincial Coordinators addressing common inquiries, challenges, and successes

*AB* Alberta, *BC* British Columbia, *CBI* capacity building intervention, *NS* Nova Scotia

### Sample size calculation

Forty-two facilities in total were required to detect a medium to large effect (0.5) in vending product health profiles with 80% power and α = 0.05 (G*Power v 3.1).

### Data collection

Primary study outcomes reported in this paper were assessed at baseline (December, 2015 – May, 2016) and 18 months later in all facilities, including: 1) Facility capacity; 2) Policy development; and 3) Food environment quality.

#### Facility capacity

Facility capacity to support the provision and promotion of healthy foods was assessed using a modified Facilities Assessment questionnaire that we have successfully used to detect differences in facility capacity in previous investigations [2, 24] (Additional file 3). Given that the current study included objective measures of the food environment, questions related to the perceived quality of the food environment were omitted. A representative from each facility answered 10 questions (response options: 0 = not in place; 1 = under development; 2 = partially in place/could be improved; 3 = fully in place) regarding their facility’s status with respect to strategic planning (e.g. policies and/or plans are in place) and communication and education (e.g. staff/volunteers receive nutrition training) for a total possible score of 30.

#### Nutrition policy development

Policy development at the facility level was assessed using one item from the Facilities Assessment questionnaire that asked whether a written nutrition policy/guideline/plan was: not in place, under development, partially in place, or fully in place (Additional file 3). Responses were dichotomized as: fully/partially in place vs under development/not in place.

#### Food environment - vending machines

In each facility, a Research Assistant used a reliable (test-retest and inter-rater reliability ≥0.88) four-step process to audit each of two randomly selected snack and beverage vending machines [24] (Additional file 4). Information on product brand, variety/type, size and flavour was recorded for each item to calculate the proportion of products classified as: Sell Most (i.e. nutrient-rich; lower in sodium, sugar and fat), Sell Sometimes (i.e. source of essential nutrients; higher in sodium, sugar, and/or fat), and Do Not Sell (i.e. energy-dense and nutrient-poor; high in sodium, sugar, and/or fat) [13], using an online automated classification tool [32]. Products were classified using BC’s food classification scheme to provide a consistent basis for cross-province comparisons.

#### Food environment - concessions

The majority of facilities had a single concession. When more than one concession was present, the one able to provide itemized sales data was audited (these data are forthcoming); otherwise a target concession was randomly selected. For each target concession, we calculated the proportion of packaged Sell Most, Sell Sometimes and Do Not Sell products as previously described, and subsequently assessed overall food environment quality using an adapted version of the Nutrition Environment Measures Survey–Restaurant reduced item audit (rNEMS-R; fast casual and fast food versions; Additional file 5) [33]. Scores on the rNEMS-R do not differ significantly from the full valid and reliable NEMS-R [34]. Higher scores (possible range for fast casual: − 4.02 to + 48.27; fast food: − 10.17 to + 51.35) indicate greater availability, supports for (e.g. signage and promotions), and lower cost of healthy options [33]. We also assessed the availability of specific ‘marker’ foods (e.g. fruit, vegetables, regular and baked potato chips, French Fries) and food preparation equipment (e.g. deep fat fryer, grill) listed in the full NEMS-R [34], along with alcohol, as indicators of the healthfulness of food environments.

### Data analysis

Descriptive characteristics were calculated as proportions, means and ranges. Repeated measures ANOVA assessed differences in facility capacity and food environments across the three conditions over time, adjusting for baseline values. Values for vending machines of the same type were averaged within facilities. The effect of time by condition was the main outcome of interest. Where there were significant main effects of time by condition, pairwise comparisons using Bonferroni correction were conducted. Associations between condition and policy development (fully/partially in place vs under development/not in place) and availability of marker foods and food preparation equipment (healthy change/maintained healthy status vs not) over time were examined using Chi-square Tests of Association.

For all comparisons, differences in outcomes between GL + CBI and GL-ONLY facilities captured the effect of the CBI, whereas comparisons of these groups with NO-GL facilities captured changes associated with provincial nutrition guidelines, with or without capacity building. All analyses were by originally assigned groups. The proportion of missing data was very low and thus a complete case analysis was conducted under a missing completely at random (MCAR) assumption, which was a reasonable assumption given reasons for missingness described below. Findings were unchanged when the five missing facility capacity scores were carried forward from baseline to follow-up. SPSS (version 24; IBM Corporation, Armonk, NY) was used for all analyses, with *p* <  0.05 indicating significant differences.

#### Sensitivity analyses

As part of the CBI, GL + CBI facilities created their own facility-specific change plans. As a result, some facilities chose to focus their efforts on improving the nutritional quality of their vending machine items, whereas others focussed on improving the nutritional quality of their concession items, rather than attempting to change both simultaneously. Sensitivity analyses therefore examined whether findings differed when only those facilities that were actively working to improve the proportion of healthy vending (*n* = 11) or concession (*n* = 7) items, respectively, were included in the analyses.

## Results

### Descriptive characteristics

All 49 facilities completed the study and provided follow-up data (note that not all facilities had snack and beverage vending machines and a concession, and some concessions did not sell packaged beverages and others did not sell packaged snacks). Five facilities did not report facility capacity scores at follow-up (citing lack of time). Two concessions closed during the course of the study. Moreover, two concessions stopped selling packaged snacks and one stopped selling packaged beverages between baseline and follow-up.

Table 2 presents baseline descriptive characteristics for facilities. At baseline, NO-GL facilities had lower capacity (Table 3) and poorer quality food environments (Tables 4 and 5) relative to facilities in guideline provinces.

**Table 2:** Facility characteristics at baseline for the Eat, Play, Live trial

Characteristic	NO-GL *n* = 17	GL-ONLY *n* = 15	GL + CBI *n* = 17
Number of facilities (*n*)			
*British Columbia*	0	7	7
*Alberta*	0	5	6
*Nova Scotia*	0	3	4
*Ontario*	17	0	0
Community size (% of facilities)^a^			
*Rural*	0	6.7	5.9
*Small population centre*	17.6	66.0	47.0
*Medium population centre*	17.6	0	5.9
*Large urban population centre*	64.7	26.7	41.2
Facility size (% of facilities)^b^			
*Small*	5.9	46.7	35.3
*Medium*	35.3	20.0	23.5
*Large*	58.8	33.3	41.2
Mean number of vending machines (range)	6.5 (1–15)	3.6 (0–11)	5.8 (0–25)
Mean number of concessions (range)	1.1 (1–2)	0.8 (0–2)	1.1 (0–4)

*GL + CBI* guideline + capacity building intervention facilities, *GL-ONLY* guideline only facilities, *NO-GL* no guideline facilities

^a^Values obtained from Statistics Canada [66] and classified according to Statistics Canada definitions [67]

^b^A small facility had at least one minor or major amenity; a medium facility had at least two amenities, one of which was major; a large facility had at least three amenities, two of which were major. Major amenities: ice rink, swimming pool, multiple courts (e.g. tennis, squash/racquetball), bowling alley, theatre (e.g. movie, performing arts). Minor amenities: community and multi-purpose room, fitness room, small specialty area such as small climbing wall or gymnastics room

**Table 3:** Change in facility capacity by intervention group in the Eat, Play, Live trial

	*n*	Mean % ideal score (SD)		Intervention effect^a^	
		Baseline	Follow-up	F	*p* value
Facility capacity subscores					
Strategic planning					
GL + CBI	16	22.9 (24.1)	60.9 (28.2)	10.050	< 0.001^b^
GL only	13	16.0 (26.2)	16.0 (25.8)		
No GL	14	6.0 (12.4)	23.2 (23.6)		
Communication and education					
GL + CBI	16	36.1 (19.8)	63.2 (27.1)	3.890	0.029^c^
GL only	13	29.1 (23.7)	30.8 (28.1)		
No GL	14	21.8 (15.0)	40.1 (19.1)		
Overall facility capacity score					
Total					
GL + CBI	16	30.8 (15.6)	62.3 (22.0)	7.908	0.001^b^
GL only	13	23.8 (21.7)	24.9 (26.1)		
No GL	14	15.5 (12.9)	33.3 (14.3)		

*GL + CBI* guideline + capacity building intervention facilities, *GL only* guideline only facilities, *No GL* no guideline facilities

At follow-up 1 GL + CBI, 2 GL-ONLY and 3 NO-GL facilities did not report facility capacity scores

^a^The intervention effect is the *p* value for the interaction of time by condition from repeated measures ANOVA

^b^Significant pairwise differences between GL + CBI and GL-ONLY (*p* < 0.01); and between GL + CBI and NO-GL (*p* < 0.01)

^c^Significant pairwise differences between GL + CBI and GL-ONLY (*p* < 0.05); and between GL + CBI and NO-GL (*p* < 0.05)

**Table 4:** Change in the proportion of snacks and beverages in vending machines by intervention group in the Eat, Play, Live trial

	*n*	Mean % (SD)		Intervention effect^a^	
		Baseline	Follow-up	F	*p* value
Beverages					
Do Not Sell					
GL + CBI	16	59.1 (17.2)	51.8 (20.1)	0.667	0.52
GL-ONLY	12	71.7 (14.1)	64.8 (20.5)		
NO-GL	17	80.8 (10.2)	79.2 (14.4)		
Sell Sometimes					
GL + CBI	16	21.4 (12.7)	24.9 (16.2)	0.043	0.96
GL-ONLY	12	14.6 (12.6)	18.7 (13.2)		
NO-GL	17	10.8 (4.4)	13.4 (9.2)		
Sell Most					
GL + CBI	16	19.4 (11.9)	23.3 (11.3)	1.596	0.22
GL-ONLY	12	13.7 (7.4)	16.5 (11.3)		
NO-GL	17	8.5 (9.8)	7.4 (9.1)		
Snacks					
Do Not Sell					
GL + CBI	14	74.0 (16.6)	45.2 (20.1)	15.629	< 0.001^b^
GL-ONLY	10	80.0 (22.4)	81.0 (22.1)		
NO-GL	12	91.1 (8.8)	90.6 (6.3)		
Sell Sometimes					
GL + CBI	14	22.4 (14.4)	43.8 (15.8)	15.529	< 0.001^b^
GL-ONLY	10	15.0 (15.2)	15.2 (16.6)		
NO-GL	12	6.0 (6.3)	6.7 (5.1)		
Sell Most					
GL + CBI	14	3.7 (4.4)	11.0 (9.0)	4.310	0.002^c^
GL-ONLY	10	4.9 (8.3)	3.8 (6.5)		
NO-GL	12	2.8 (3.4)	2.7 (4.7)		

*GL + CBI* guideline + capacity building intervention facilities, *GL-ONLY* guideline only facilities, *NO-GL* no guideline facilities

A total of 83 beverage (52% of all beverage machines; *n* = 2110 products) and 58 snack vending machines (78% of all snack machines; *n* = 1983 products) were audited at baseline. A total of 79 beverage (54% of all beverage machines; *n* = 2106 products) and 59 snack vending machines (80% of all snack machines; *n* = 2067 products) were audited at follow-up

^a^The intervention effect is the *p* value for the interaction of time by condition from repeated measures ANOVA

^b^Significant pairwise differences between GL + CBI and GL-ONLY (p < 0.01); and between GL + CBI and NO-GL (p < 0.001)

^c^Non-significant trend toward pairwise differences between GL + CBI and NO-GL (*p* = 0.08)

**Table 5:** Change in the proportion of packaged snacks and beverages and in the overall quality of the concession food environment by intervention group in the Eat, Play, Live trial

	*n*	Mean % (SD)		Intervention effect^a^	
		Baseline	Follow-up	F	*p* value
Packaged beverages					
Do Not Sell					
GL + CBI	9	73.1 (9.4)	74.2 (9.5)	0.478	0.62
GL-ONLY	11	71.8 (13.6)	73.7 (9.8)		
NO-GL	16	80.3 (9.2)	85.3 (8.4)		
Sell Sometimes					
GL + CBI	9	19.0 (10.6)	20.3 (10.7)	0.895	0.42
GL-ONLY	11	20.6 (15.9)	18.2 (11.2)		
NO-GL	16	16.6 (8.4)	11.8 (6.9)		
Sell Most					
GL + CBI	9	7.9 (3.5)	5.5 (2.9)	1.805	0.18
GL-ONLY	11	7.6 (6.5)	8.1 (8.8)		
NO-GL	16	3.2 (3.3)	2.9 (3.9)		
Packaged snacks					
Do Not Sell					
GL + CBI	7	83.4 (18.3)	69.1 (21.6)	1.550	0.23
GL-ONLY	11	74.9 (25.6)	78.0 (12.4)		
NO-GL	16	93.6 (7.1)	93.7 (9.3)		
Sell Sometimes					
GL + CBI	7	15.1 (17.8)	26.5 (16.9)	1.213	0.31
GL-ONLY	11	21.0 (27.4)	17.9 (9.8)		
NO-GL	16	5.6 (6.3)	5.4 (9.2)		
Sell Most					
GL + CBI	7	1.5 (2.6)	4.4 (10.2)	0.529	0.59
GL-ONLY	11	4.1 (6.1)	4.1 (5.5)		
NO-GL	16	0.8 (1.8)	0.9 (2.1)		
rNEMS-R Scores^a^					
		Mean (SD) [% ideal]		Intervention effect^b^	
Fast casual score		Baseline	Follow-up	F	*p* value
GL + CBI	11	15.5 (9.0)	17.7 (6.7)	2.675	0.08
		[37.3%]	[41.5%]		
GL-ONLY	11	15.5 (8.0)	13.2 (5.3)		
		[37.3%]	[32.9%]		
NO-GL	16	8.4 (3.5)	7.3 (3.8)		
		[23.8%]	[21.6%]		
Fast food score					
GL + CBI	11	14.7 (8.4)	17.5 (7.2)	10.958	< 0.001^c^
		[40.4%]	[45.0%]		
GL-ONLY	11	14.8 (6.5)	14.1 (5.1)		
		[40.6%]	[39.4%]		
NO-GL	16	9.2 (4.3)	4.0 (4.4)		
		[31.5%]	[23.0%]		

*GL + CBI* guideline + capacity building intervention facilities, *GL-ONLY* guideline only facilities, *NO-GL* no guideline facilities, *rNEMS-R* Nutrition Environment Measures Survey–Restaurant reduced item audit

A total of 40 concessions were audited at baseline and 38 concessions were audited at follow-up. 8 facilities did not have a concession, 1 could not be audited at baseline or follow-up and 2 concessions closed between baseline and follow-up. In addition, 1 concession no longer sold packaged beverages and 2 no longer sold packaged snacks at follow-up. A total of 756 packaged beverages and 772 packaged snacks were audited at follow-up

^a^Higher scores (possible range for fast casual: - 4.02 to + 48.27; fast food: −10.17 to + 51.35) indicate greater availability, supports for, and lower cost of healthy options

^b^The intervention effect is the p value for the interaction of time by condition from repeated measures ANOVA

^c^Significant pairwise differences between GL + CBI and NO-GL (p < 0.001); and between GL-ONLY and NO-GL (p < 0.01)

### Facility capacity

There was a significant effect of time (F = 28.618; *p* <  0.001) and time by condition (F = 7.908; *p* = 0.001) for facility capacity scores (Table 3). Overall facility capacity (% ideal score) increased from 30.8 ± 15.6% to 62.3 ± 22.0% in GL + CBI facilities compared to GL-ONLY (23.8 ± 21.7% to 24.9 ± 26.1%; *p* <  0.01) and NO-GL facilities (15.5 ± 12.9% to 33.3 ± 14.3%; p <  0.01).

### Nutrition policy development

Between baseline and follow-up, 17.6% of GL + CBI facilities developed new written facility nutrition policies whereas no GL-ONLY or NO-GL facilities developed new written policies (X^2^ = 6.015, *p* = 0.049).

### Food environment – vending machines

There was a significant effect of time for Do Not Sell vending beverages (F = 4.951, *p* = 0.03). There were no significant time by condition effects for vending beverages (Table 4).

There was a significant effect of time for Do Not Sell (F = 13.687; p = 0.001) and Sell Sometimes (F = 15.995; p <  0.001) vending snacks. There were also significant time by condition effects for vending snacks (Table 4). The proportion of Do Not Sell snacks in GL + CBI facilities declined from 74.0 ± 16.6% to 45.2 ± 20.1% compared to GL-ONLY (80.0 ± 22.4% to 81.0 ± 22.1%; p <  0.01) and NO-GL facilities (91.1 ± 8.8% to 90.6 ± 6.3%; *p* < 0.001). The proportion of Sell Sometimes snacks in GL + CBI facilities increased from 22.4 ± 14.4% to 43.8 ± 15.8% compared to GL-ONLY (15.0 ± 15.2% to 15.2 ± 16.6%; *p* < 0.01) and NO-GL facilities (6.0 ± 6.3% to 6.7 ± 5.1%; p < 0.001). For Sell Most snacks the overall time by condition effect was significant (*p* = 0.002), however post-hoc analyses revealed no significant pairwise differences, although there was a trend toward an increase in the proportion of Sell Most snacks in GL + CBI (3.7 ± 4.4% to 11.0 ± 9.0%) relative to NO-GL facilities (2.8 ± 3.4 to 2.7 ± 4.7; *p* = 0.08).

### Food environment – concessions

There were no significant effects of time or time by condition for packaged snacks and beverages in concessions (Table 5).

There were no significant effects of time for rNEMS-R scores. There were significant time by condition effects for the fast food version of the rNEMS-R score (Table 5). rNEMS-R scores increased in GL + CBI (14.7 ± 8.4 to 17.5 ± 7.2), compared to NO-GL facilities (9.2 ± 4.3 to 4.0 ± 4.4; p < 0.001), and declined in NO-GL (9.2 ± 4.3 to 4.0 ± 4.4) relative to GL-ONLY facilities (14.8 ± 6.5% to 14.1 ± 5.1; *p* < 0.01). These changes trended in a similar direction using the fast casual version of the rNEMS-R score, but were not statistically significant (p = 0.08).

GL + CBI facilities exhibited the healthiest mix of marker foods over time. Nearly three-quarters increased/maintained provision of fruits and vegetables, nearly half offered a healthier mix of potato chips (i.e. more baked and fewer regular varieties) and more than one-third increased/maintained provision of healthy main and side dishes (Table 6).

**Table 6:** Availability of marker foods and food preparation equipment in the Eat, Play, Live trial

	Proportion of facilities that made healthy changes or maintained a healthy status from baseline to follow-up			*p* value
	GL + CBI (n = 11)	GL-ONLY (n = 11)	NO-GL (*n* = 16)	
Foods and beverages				
Low fat milk	9.1%	18.2%	0%	0.22
100% juice	90.9%	100%	100%	0.28
Whole grain bread	72.7%	63.6%	37.5%	0.16
White bread	9.1%	9.1%	18.8%	0.69
French fries	36.4%	45.5%	31.3%	0.75
Potato chips^a^	45.5%	27.3%	0%	0.015
Fruit	72.7%	81.8%	12.5%	< 0.001
Vegetables	72.7%	36.4%	6.3%	0.002
High fat side	27.3%	18.2%	25.0%	0.87
Healthy main dish	36.4%	9.1%	0%	0.021
Healthy main dish salad	36.4%	9.1%	0%	0.021
Food preparation equipment				
Deep fat fryer	36.4%	45.5%	31.3%	0.75
Griddle	45.5%	45.5%	81.3%	0.08
Grill	45.5%	9.1%	18.8%	0.11
Oven	63.6%	45.5%	56.3%	0.69

*GL + CBI* guideline + capacity building intervention facilities, *GL-ONLY* guideline only facilities, *NO-GL* no guideline facilities, *rNEMS-R* reduced Nutrition Environment Measures Survey - Restaurant

Healthy change/maintenance scores were calculated using Chi-square statistics based on the presence or absence of the item at baseline and follow-up (i.e. a healthy change was not selling a healthy item at baseline but selling it at follow-up or selling an unhealthy item at baseline and not selling it at follow-up; maintenance of a healthy status was selling a healthy item at baseline and follow-up)

A total of 40 concessions were audited at baseline and 38 concessions were audited at follow-up. 8 facilities did not have a concession, 1 could not be audited at baseline or follow-up and 2 concessions closed between baseline and follow-up

^a^Potato chips combines information about the change in availability of baked and regular potato chips from baseline to follow-up. For facilities that sold regular and baked, or only baked potato chips at baseline, a healthy change was defined as no longer selling any potato chips at follow-up. For facilities that sold only regular potato chips at baseline a healthy change was defined as no longer selling regular chips and/or adding baked chips at follow-up. Selling baked chips at baseline and follow-up was classified as maintaining a healthy status

### Sensitivity analyses

Findings were unchanged in sensitivity analyses except in one respect, as there was a significant effect of time by condition for rNEMS-R fast casual scores. rNEMS-R scores increased in GL + CBI (13.8 ± 8.0% to 16.7 ± 6.1%) compared to NO-GL facilities (8.4 ± 3.5 to 7.3 ± 3.8; p < 0.01), and contrary to expectations declined in GL-ONLY (15.5 ± 8.0 to 13.2 ± 5.3) relative to NO-GL facilities (8.4 ± 3.5 to 7.3 ± 3.8; p < 0.01).

## Discussion

The EPL trial leveraged differences in provincial nutrition guidelines for recreation and sport facilities as an opportunity to examine the impact of an 18 month CBI in enhancing implementation of nutrition guidelines. We also examined whether nutrition guidelines were associated with positive changes over time. Primary study outcomes were facility capacity, policy development and food environment quality. Although facilities in guideline provinces started at a healthier place relative to those in a province without nutrition guidelines (unpublished data), provincial nutrition guidelines alone did not provoke positive changes during this 18 month study. Significant improvements in facility capacity, policy development, and in food environment quality (vending and concession) only occurred in facilities that were both exposed to nutrition guidelines and participated in an actively delivered CBI. Thus, ongoing capacity building is needed to realize the positive potential of voluntary nutrition guidelines. Nevertheless, food environments remained overwhelmingly unhealthy in all cases, suggesting additional scope to enhance implementation of nutrition guidelines.

Effective nutrition policies have been defined as those that lead to positive changes in food, social and informational environments [35]. Improvements to food environments may, in turn, improve children’s dietary intake [5, 6]. To be effective in changing food environments and dietary intake however, policies must be implemented. The difficulty of achieving high levels of policy implementation can be substantial [36–39], particularly when policies are voluntary [2, 17]. Indeed, although guideline facilities had healthier food environments than no guideline facilities at baseline (unpublished observations), their food environments remained overwhelmingly unhealthy, demonstrating a failure to fully implement nutrition guidelines. Moreover, there were no positive changes in the food environments of guideline only facilities over the course of the study. This suggests that provincial nutrition guidelines may have prompted early positive change in recreation and sport facility food environments, but that without ongoing support, the change process stagnated. It is possible that managers perceived they were doing enough, and that further change was not needed. Having mandated policies, with clear standards of accountability, could help to ensure managers understand what constitutes meaningful change to improve food environments.

Organizational capacity building enhanced implementation of voluntary provincial nutrition guidelines in recreation and sport facilities across three provinces. These changes were realized at a relatively low cost of approximately $2950/facility (CAD). The most significant changes observed were with respect to the quality of snack provision within vending machines, with significant declines in unhealthy, and significant increases in healthier products in GL + CBI facilities. In a pilot study of an early version of the CBI we also observed positive shifts in the provision of vending products over a shorter 8 month time frame [2]. Although results for concession snacks trended in similar directions, the variability among facilities was high and thus there were no significant changes. Given that some facilities did not have concessions, this analysis may also have been underpowered. A nearly 5% improvement in the overall quality of concession food environments was, however, observed in GL + CBI facilities, along with positive shifts in the proportion of facilities offering healthy marker foods, including fruits, vegetables, healthy main dishes and sides. The latter findings may be more informative than changes in the proportional availability of healthy and unhealthy concession items, as items sold in concessions are not typically prominently displayed as they are in vending machines (i.e. most items are simply listed in text on menu boards and/or a single facing of each item is on display). As such the proportionality metric may be a less informative indicator of food environment quality in concessions. The only other RCT conducted in a similar context also found positive shifts in the provision of fruits and vegetables following a multi-component canteen intervention, albeit on the basis of self-reported data [40]. However, in agreement with our findings, there were no significant changes in availability of non-sugar-sweetened drinks. It is possible that managers were attempting to limit their risk through step-wise changes and perceived greater opportunity to improve the healthfulness of meals and snacks.

Organizational capacity building is an evidence-based strategy that can support organizations in mobilizing policy into practice by ensuring adequate resources, supportive structures and workforce skills are available [41]. The EPL CBI succeeded in building facility capacity, as evidenced by significant increases in capacity scores in GL + CBI facilities (from 31 to 62% of ideal score). These increases in facility capacity coincided with significant increases in nutrition policy development at a facility level and in the quality of food environments. Although smaller, but non-significant increases in capacity were also observed in NO-GL facilities (from 16 to 33% of ideal score), there were no concurrent changes in policy development or food environment quality. This may suggest a threshold effect. NO-GL facilities may not have reached the critical capacity threshold capable of yielding significant positive change. Our findings clearly show that one reason why provincial nutrition guidelines have not been fully implemented in recreation and sport facilities is because facilities lack capacity to do so. That scores were just 62% of ideal in GL + CBI facilities suggests there may be additional scope to improve food environments through further capacity building. Alternatively, that large changes in facility capacity did not translate into larger positive changes in food environments may also indicate that capacity building alone is not sufficient to create truly healthy food environments in the context of voluntary nutrition guidelines, and where cultural norms promote the opposite. A multi-component implementation intervention in Australian sports clubs that included several analogous implementation strategies also found positive, albeit limited improvements in food environments [42].

Policy-focused and policy-supported interventions have proven effective in improving population health [43–46] and therefore the CBI encouraged development of nutrition policies at a facility level. Whereas no comparison facilities developed written nutrition policies during the course of the study, nearly one-fifth of GL + CBI facilities did. The deliberative processes involved in formalizing written policies can assist facilities to clarify and formalize their core values [47], and may therefore have stimulated change to food environments within these facilities. Moreover, policies codify change and often survive transitions in leadership, which may enhance the sustainability of change [48]. The presence of facility-specific nutrition policies at baseline was associated with healthier food environments (unpublished observations). We therefore expect that provincial and local policies might be mutually reinforcing over time.

Despite significant improvements in some aspects of facilities’ food environments, they nevertheless remained overwhelmingly unhealthy. Several factors in combination may explain these findings. First, change takes time. Unhealthy food and drink are deeply embedded within the culture of sport [4, 19], and change will require both initiative-taking on the part of mangers to supply healthier foods, and a demand ‘pull’ from consumers to purchase these items. As gate-keepers of food environments in recreation and sport, managers are crucial to realizing such change. Through incrementally improving the nutritional quality of foods on offer, managers can help to gradually accustom patrons to seeing and purchasing healthier foods [4]. Second, although the CBI addressed important barriers previously identified in this context (e.g. perceived lower profitability of healthier items, limited knowledge and skills to implement nutrition guidelines, minimal stakeholder buy-in and cooperation [1, 2, 4, 18, 19]), the strategies presented in the CBI may not have been sufficient to fully overcome them. Moreover, the CBI did not address barriers at the patron level, as parents admit to purchasing unhealthy foods for their children because factors such as convenience, cost and child preferences sometimes take precedence over health [49–52]. More than 80 distinct capacity building elements have been identified in the literature [53]. Thus, greater tailoring of the CBI to the needs and contexts of particular facilities may also be required [54]. Third, EPL focussed on building capacity at the organizational rather than at the individual practitioner level, and these two types of capacity are mutually reinforcing [41]. Building capacity at both levels may provoke greater change. Finally, low availability of healthy, shelf-stable, tasty products in the marketplace likely limited the extent of change that facilities could make [18].

### Implications

Unhealthy food environments may promote correspondingly unhealthy dietary patterns, particularly among children who have less control over their surroundings [5]. It is therefore concerning that the majority of recreation and sport facilities had unhealthy food environments regardless of the presence of voluntary nutrition guidelines. This is consistent with our previous findings in recreation facilities in Canada [1, 2, 4, 16], and with studies internationally in a variety of sports settings [40, 55–59]. As identified in a recent systematic review, there is almost no knowledge of how to improve policy implementation in sports settings, with just three controlled studies meeting inclusion criteria [60]. EPL demonstrated how principles related to organizational capacity building can be successfully operationalized to improve implementation of nutrition guidelines in recreation and sport facilities at a relatively low cost. Findings provide key data for policy makers seeking to enhance implementation of voluntary guidelines, which may be more politically palatable than mandated policy. Even the small changes observed here can support healthier dietary patterns at a population-level, and may portend greater benefits in future by initiating processes of change. Dietary patterns established in childhood and adolescence may be enduring [61, 62], and thus reengineering food environments in recreation and sport facilities remains a high priority. Ultimately, mandated, monitored and enforced nutrition policies, alongside change in social norms, may be required to achieve full implementation of nutrition guidelines.

### Strengths and limitations

The EPL trial was designed to maximize both internal and external validity by embedding a RCT within a natural experiment conducted across provinces at different stages of guideline implementation. Given the heterogeneity in provincial contexts, stages of guideline implementation, facility types and sizes, and the remarkable consistency in the barriers to change encountered in previous Canadian [2, 4, 17–19, 63] and international studies [52, 64, 65], we expect the generalizability of findings to be relatively high. Nevertheless, the poor representation of rural and medium population centres remains a limitation.

The CBI had a strong theoretical grounding in a linking system approach. It incorporated evidence-based elements identified in the wider capacity-building literature and also targeted specific barriers previously encountered in this setting. It also mimicked a real-world context by relying largely on existing supports and resources to support recreation stakeholders in the change process, and by having facilities create and execute their own contextualized action plans. These features may enhance the sustainability of the changes observed here, however, we do not have data in this respect. Ultimately, the sustainability of change will likely depend on whether and to what extent any changes influence profitability.

Risk of bias was minimized by randomization, objective assessment of food environments by trained Research Assistants, and by the lack of attrition and little missing data. However, limitations remain, as food environments were assessed at a single point in time, although previous data indicate that the contents of vending machines do not typically vary substantially from week to week [24]. It was not possible to comprehensively measure all aspects of food environments, and thus facilities may have made changes in other areas that were not captured here. This could have had unintended consequences, whereby managers focussed on making changes in the areas we assessed, while letting others go unaddressed. Our analyses were underpowered to detect small changes, thereby increasing the chance of type 2 errors. With a larger sample size, some non-significant trends in the data may have reached statistical significance. Guideline facilities were randomly assigned to GL + CBI or GL-ONLY conditions, however facilities could not be randomly assigned to the NO-GL condition. However, as all facilities were publicly funded, they did not self-select to locate in a particular province and therefore assignment to condition occurred through what was essentially a random process. It is possible that facilities with a greater interest in nutrition may have been more likely to participate in the study. Finally, the impact of changes to food environments on children’s food intake was not assessed.

## Conclusions

Findings from the EPL trial show the potential to leverage a capacity building approach that relies largely on existing resources and supports to protect government investments in developing and disseminating voluntary nutrition guidelines for recreation and sport facilities. Significant improvements in facility capacity, policy development and food environment quality occurred in recreation and sport facilities that were both exposed to nutrition guidelines and that participated in an actively delivered CBI. Outcomes did not improve significantly in facilities that were only passively or not at all exposed to provincial nutrition guidelines. Although facilities in guideline provinces started at a healthier place relative to those in a province without nutrition guidelines, without ongoing capacity building the change process ultimately stagnated. Food environments remained overwhelmingly unhealthy in all facilities, however, suggesting additional scope to enhance implementation of nutrition guidelines through more extensive capacity building. Alternatively, mandated, monitored and enforced nutrition policies may be required to ensure that food environments in recreation and sport facilities support, and do not undermine, children’s health.

## ADDITIONAL FILES

Additional file 1: CONSORT checklist. (PDF 147 kb)

Additional file 2: TIDieR checklist. (PDF 206 kb)

Additional file 3: Facilities Assessment questionnaire. (PDF 219 kb)

Additional file 4: Vending audit. (PDF 390 kb)

Additional file 5: Nutrition Environment Measures Survey–Restaurant reduced item audit. (PDF 311 kb)

## Data Availability

The datasets used and analysed during the current study are available from the corresponding author on reasonable request.

## Abbreviations

AB: Alberta
BC: British Columbia
CBI: Capacity building intervention
EPL: Eat, Play, Live
GL + CBI: Guideline + capacity building intervention
GL-ONLY: Guideline only
NEMS-R: Nutrition Environment Measures Survey–Restaurant
NO-GL: No guideline
NS: Nova Scotia
RCT: Randomized controlled trial
rNEMS-R: Nutrition Environment Measures Survey–Restaurant reduced item audit
