# Supplementary material for: Eat, play, live: a randomized controlled trial within a natural experiment examining the role of nutrition policy and capacity building in improving food environments in recreation and sport facilities
Source: Int J Behav Nutr Phys Act. 2019 Jun 25;16:51. doi: 10.1186/s12966-019-0811-8 (PMC6593504; doi:10.1186/s12966-019-0811-8)
Supplement: Supplementary file 3 — Facilities Assessment questionnaire. (PDF 219 kb) [file 12966_2019_811_MOESM3_ESM.pdf]

ADDITIONAL FILE 2

A. FACILITIES ASSESSMENT (BC, AB, NS)

☐ **Baseline:** Date___________________

☐ **Follow-up:** Date

Completing this survey implies your consent. You may withdraw your consent at any time without any consequences or any explanation. If you do withdraw from the study your data will not be used.

About Opinio. The survey is constructed and housed on the Dalhousie University Opinio survey site. Your responses to the survey will be stored on a secure Dalhousie webserver that is under the control of the Dalhousie University Networks and Systems Department. Systems are in place to limit access only to Network and Systems staff who provide operating systems level support (i.e. ensuring the survey and associated databases are working properly) and the research staff for Eat, Play, Live. Access to the server and hence the survey is password protected and data are encrypted. The level of security is at or above a level that would be recommended for the transmission of sensitive information such as credit card details. Once the survey has been completed the data will be exported to a local area network drive at the University of Victoria. The local area network drive is password protected. Confidentiality and privacy will be maintained by only giving data access to the staff working on the project.

This assessment will help you identify and plan improvements in your recreation facility's overall food environment. You will be assessing the following categories for your organization:

- **A.** Strategic Planning
- **B.** Communication and Education

Work through the assessment checklists on the following pages and identify how closely each statement best describes your facility(s)'s current stage of development. <u>You can save your answers to the questions by clicking the 'save' button and once you have completed all of the questions, click 'finish' to submit the survey.</u>

This assessment may be completed by just one person or in a group format. To begin, please fill out the following information:

<table>
<tr><td rowspan="7">

Community: ______________________________

Facility Name: ___________________________

This Assessment was completed by:

☐ **Recreation staff member:** ________________

☐ **Consultant**:______________________________

☐ **Committee:** ______________________________

Other (please specify):

____
____
____
____
____
____

</td><td colspan="5">What type of food sales outlets are in the facility(s)?</td></tr>
<tr><td></td><td>Publically operated and/or funded</td><td>Privately operated by contractor</td><td>Voluntarily operated</td><td><u>N/A</u></td></tr>
<tr><td>Cafeteria</td><td>☐</td><td>☐</td><td>☐</td><td>☐</td></tr>
<tr><td>Snack Bar/Concession</td><td>☐</td><td>☐</td><td>☐</td><td>☐</td></tr>
<tr><td>Snack Vending Machines</td><td>☐</td><td>☐</td><td>☐</td><td>☐</td></tr>
<tr><td>Beverage Vending Machines</td><td>☐</td><td>☐</td><td>☐</td><td>☐</td></tr>
<tr><td>Fundraising (e.g. bake sales or BBQs)</td><td>☐</td><td>☐</td><td>☐</td><td>☐</td></tr>
</table>

STRATEGIC PLANNING

	Fully in Place	Partially in Place/ Could be Improved	Under Development	Not in Place
a) *A 'Healthy Food' Committee has been formed* <u>*in your facility.*</u>	**3**	**2**	**1**	**0**
b) *A 'Healthy Food' policy/guideline/plan has been written and approved by decision makers for your facility. *SEE NOTE BELOW*	**3**	**2**	**1**	**0**
c) *The facility has dedicated resources and/or assigned responsibility for the plan and/or policy.*	**3**	**2**	**1**	**0**
d) *The facility's healthy choices goals are monitored and evaluated annually.*	**3**	**2**	**1**	**0**
**Column Totals →**				
Add the total points from the column totals**= Category Total →**				
(Category Total ÷ 12 x 100)= **Category Score →** This represents how close you facility comes to ideal in the strategic planning category.				%

<u>* NOTE: IF YOU HAVE A 'HEALTHY FOOD' POLICY/GUIDELINE/PLAN IN PLACE, PLEASE SEND A COPY TO YOUR EAT, PLAY LIVE PROVINCIAL COORDINATOR.</u>

Please list or explain any other work that your facility is doing in terms of strategic planning for healthy choices (e.g. setting goals, <u>meeting with community stakeholders</u>, etc.).

COMMUNICATION & EDUCATION

	Fully in Place	Partially in Place/ Could be Improved	Under Development	Not in Place
a) Training on nutrition and healthy eating is provided for staff and volunteers.	**3**	**2**	**1**	**0**
b) Food Safety (e.g. Food Safe, Food Hygiene, etc.) training is provided for staff and volunteers.	**3**	**2**	**1**	**0**
c) The public is made aware of the facility's healthy eating initiatives. (e.g. newsletter, website, posters)	**3**	**2**	**1**	**0**
d) Workshops, classes or other educational opportunities in relation to healthy eating are offered regularly.	**3**	**2**	**1**	**0**
e) Staff members are supported making healthy eating changes to the facility.	**3**	**2**	**1**	**0**
f) Programs or initiatives are underway to educate children or the public about healthy food choices.	**3**	**2**	**1**	**0**
**Column Totals →**				
Add the total points from the column totals= **Category Total →**				
(Category Total ÷ 18 x 100)= **Category Score →** This represents how close you facility comes to ideal in the communication and education strategy.				%

Please list or explain any other work that your facility is doing to increase communication and/or education for healthy food choices.

FACILITIES ASSESSMENT FINAL SCORE

Community: ____________________________

Date: ___________________________________

- Transfer the category scores from the bolded boxes for each of the three assessment areas into the tally box below.
- Compare with future, annual re-assessments to track your progress.
- Be sure to save this record of your facility's status.

Example: (6/12 = 50%)

CATEGORY	Score	Out of	Percent
**Strategic Planning**		**12**	
**Communication & Education**		**18**	
Add top 2 scores for the **TOTAL →**		**30**	

This score represents how close your healthy choice facility comes to ideal. Use this percentage to assess your progress from year to year.
