# Supplementary material for: Eat, play, live: a randomized controlled trial within a natural experiment examining the role of nutrition policy and capacity building in improving food environments in recreation and sport facilities
Source: Int J Behav Nutr Phys Act. 2019 Jun 25;16:51. doi: 10.1186/s12966-019-0811-8 (PMC6593504; doi:10.1186/s12966-019-0811-8)
Supplement: Supplementary file 4 — Vending audit. (PDF 390 kb) [file 12966_2019_811_MOESM4_ESM.pdf]

ADDITIONAL FILE 3

B. VENDING AUDIT

☐ **Baseline:** Date_______________

☐ **Follow-up:** Date

This audit will require the following steps:

STEP 1: Complete a list of all vending machines in the facility. Use Random.org to choose machines to audit.

STEP 2: Create a product stock list for 2 dry snack, 2 cold beverage, 1 refrigerated snack (if available) & 1 frozen snack machine (if available).

STEP 3: Rate the 2 dry snack, 2 cold beverage, 1 refrigerated snack & 1 frozen snack machines using the Brand Name Food List. Also rate the products in these 4-6 machines using your own Province's nutritional guidelines.

Community: ____________________________ **Facility audited:** _________________________________

Date: ___________________________________ ____

This Assessment was completed by the following (check all that apply and fill in name):

□ **Provincial coordinator:** ____

□ **Research assistant:**____

□ **Other (please specify):** ____

VENDING AUDIT STEP 1: VENDING MACHINE INVENTORY

a) Tour the facility and record the type and location of all of the food and beverage machines in the table below. Based on your description of the location, you (or someone else) will need to be able to identify the exact machine in 18 months. Use left-middle-right indications for machines standing next to each other in the same location (E.g. ABC Rec. Facility, lobby, right). After you have created the list, number the cold beverage machines 1b, 2b, 3b, etc., the dry snack machines 1s, 2s, 3s, etc. refrigerated snack machines 1r, 2r, 3r, etc , the frozen machines 1f, 2f, 3f, etc. and the cold beverage machines 1b, 2b, 3b etc. Just write the word ‘other’ beside the hot beverage and other machines. Count candy dispensers as ‘other’.

**#**	**Snack**			**Beverage**		**Other (describe)**	**Location**	**Assigned for Audit**
	‘dry’ snack	refrig-erated	frozen	cold	hot			✓
sample	✓						Skate shop lobby (3rd from L in bank of 5)	

#	Snack			Beverage		Other (describe)	Location	Assigned for Audit ✓
	'dry' snack	refrig-erated	frozen	cold	hot			
sample	✓						Skate shop lobby (3rd from L in bank of 5)	

b) Tally up the number of machines in the facility that fall into each category:

Total # of dry snack machines (number as 1s, 2s, 3s, etc.) = ___________

Total # of refrigerated snack machines (number as 1r, 2r, 3r, etc.) = ___________

Total # of frozen (ie. Ice cream) machines (number as 1f, 2f, 3f, etc.) = ___________

Total # of cold beverage machines (number as 1b, 2b, 3b, etc.) = ___________

Total # of hot beverage machines (designate as 'other') = ___________

Total # of other food/beverage machines (designate as 'other') = ___________ (describe ________________________________)

Total # of vending machines **=** ___________

c) Once you have identified and numbered all of the vending machines, use the Random.org number generator to choose 2 dry snack, 2 cold beverage, 1 refrigerated snack (if available) and 1 frozen snack machine (if available) for auditing. Put a check mark beside the chosen machines in the 'Assigned for audit' column in the table.

VENDING AUDIT STEP 2: VENDING STOCK LIST

Record the code slot location by its code in the machine. Then identify the product that is in each slot according to the company that produces the product, the product's name, flavour, size and current selling price. Check the Product Binder to see if the item is on one of our food lists and ✓ 'In Product Binder?' if it is. If an item is NOT in Product Binder, get photos of it so you can find the nutrition information later.

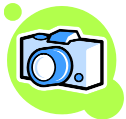

Along with each stock list please **include a photo or two** of the machine that shows its current products and promotional images.

Vending Machine #1 (specify machine location so you can re-audit after changes have been made to evaluate) ______________________

____

Machine Type: ☐ Snack ☐ Beverage ☐ Frozen ☐ Refrigerated

Vending Machine Purpose: ☐ Cafeteria ☐ Fundraising ☐Other

Vendor Company Name: ____________________________

**Slot Location** (e.g. A1 or "top left" etc.)	**Company Name** (e.g. Pepsi, Lays, Doritos etc)	**Product Name & Flavour** (e.g. Gatorade-Berry Blast, Doritos Chips-Salsa, Mars Bar)	**Size** (ml or g)	**Price** ($)	**Healthy Choice Symbol** (e.g. heart ♥ or check ✓)	**In Product Binder?** ✓
A1						

(VENDING AUDIT Step 2 – Machine #1 …)

**Slot Location** (e.g. A1 or "top left" etc.)	**Company Name** (e.g. Pepsi, Lays, Doritos etc)	**Product Name & Flavour** (e.g. Gatorade-Berry Blast, Doritos Chips-Salsa, Mars Bar)	**Size** (ml or g)	**Price** ($)	**Healthy Choice Symbol** (e.g. heart ♥ or check ✓)	**In Product Binder?** ✓

VENDING AUDIT STEP 2: VENDING STOCK LIST

Record the code slot location by its code in the machine. Then identify the product that is in each slot according to the company that produces the product, the product's name, flavour, size and current selling price. Check the Product Binder to see if the item is on one of our food lists and ✓ 'In Product Binder?' if it is. If an item is NOT in Product Binder, get photos of it so you can find the nutrition information later.

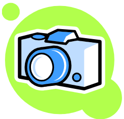

Along with each stock list please **include a photo or two** of the machine that shows its current products and promotional images.

Vending Machine #2 (specify machine location so you can re-audit after changes have been made to evaluate) ______________________

____

Machine Type: ☐ Snack ☐ Beverage ☐ Frozen ☐ Refrigerated

Vending Machine Purpose: ☐ Cafeteria ☐ Fundraising ☐ Other

Vendor Company Name: ____________________________

**Slot Location** (e.g. A1 or "top left" etc.)	**Company Name** (e.g. Pepsi, Lays, Doritos etc)	**Product Name & Flavour** (e.g. Gatorade-Berry Blast, Doritos Chips-Salsa, Mars Bar)	**Size** (ml or g)	**Price** ($)	**Healthy Choice Symbol** (e.g. heart ♥ or check ✓)	**In Product Binder?** ✓
A1						

(VENDING AUDIT Step 2 – Machine #2 …)

**Slot Location** (e.g. A1 or "top left" etc.)	**Company Name** (e.g. Pepsi, Lays, Doritos etc)	**Product Name & Flavour** (e.g. Gatorade-Berry Blast, Doritos Chips-Salsa, Mars Bar)	**Size** (ml or g)	**Price** ($)	**Healthy Choice Symbol** (e.g. heart ♥ or check ✓)	**In Product Binder?** ✓

VENDING AUDIT STEP 2: VENDING STOCK LIST

Record the code slot location by its code in the machine. Then identify the product that is in each slot according to the company that produces the product, the product's name, flavour, size and current selling price. Check the Product Binder to see if the item is on one of our food lists and ✓ 'In Product Binder?' if it is. If an item is NOT in Product Binder, get photos of it so you can find the nutrition information later.

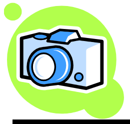

Along with each stock list please **include a photo or two** of the machine that shows its current products and promotional images.

Vending Machine #3 (specify machine location so you can re-audit after changes have been made to evaluate) ________________________

____

Machine Type: ☐ Snack ☐ Beverage ☐ Frozen ☐ Refrigerated

Vending Machine Purpose: ☐ Cafeteria ☐ Fundraising ☐ Other

Vendor Company Name: ______________________________

**Slot Location** (e.g. A1 or "top left" etc.)	**Company Name** (e.g. Pepsi, Lays, Doritos etc)	**Product Name & Flavour** (e.g. Gatorade-Berry Blast, Doritos Chips-Salsa, Mars Bar)	**Size** (ml or g)	**Price** ($)	**Healthy Choice Symbol** (e.g. heart ♥ or check ✓)	**In Product Binder?** ✓
A1						

(VENDING AUDIT Step 2 – Machine #3 …)

**Slot Location**<br>(e.g. A1 or “top left” etc.)	**Company Name**<br>(e.g. Pepsi, Lays, Doritos etc)	**Product Name & Flavour**<br>(e.g. Gatorade-Berry Blast, Doritos Chips-Salsa, Mars Bar)	**Size**<br>(ml or g)	**Price**<br>($)	**Healthy Choice Symbol**<br>(e.g. heart ♥ or check ✓)	**In Product Binder?**<br>✓

VENDING AUDIT STEP 2: VENDING STOCK LIST

Record the code slot location by its code in the machine. Then identify the product that is in each slot according to the company that produces the product, the product's name, flavour, size and current selling price. Check the Product Binder to see if the item is on one of our food lists and ✓ 'In Product Binder?' if it is. If an item is NOT in Product Binder, get photos of it so you can find the nutrition information later.

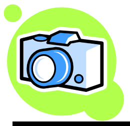

Along with each stock list please **include a photo or two** of the machine that shows its current products and promotional images.

Vending Machine #4 (specify machine location so you can re-audit after changes have been made to evaluate) ______________________

____

Machine Type: ☐ Snack ☐ Beverage ☐ Frozen ☐ Refrigerated

Vending Machine Purpose: ☐ Cafeteria ☐ Fundraising ☐Other

Vendor Company Name: ____________________________

**Slot Location** (e.g. A1 or "top left" etc.)	**Company Name** (e.g. Pepsi, Lays, Doritos etc)	**Product Name & Flavour** (e.g. Gatorade-Berry Blast, Doritos Chips-Salsa, Mars Bar)	**Size** (ml or g)	**Price** ($)	**Healthy Choice Symbol** (e.g. heart ♥ or check ✓)	**In Product Binder?** ✓
A1						

(VENDING AUDIT Step 2 – Machine #4 …)

**Slot Location** (e.g. A1 or "top left" etc.)	**Company Name** (e.g. Pepsi, Lays, Doritos etc)	**Product Name & Flavour** (e.g. Gatorade-Berry Blast, Doritos Chips-Salsa, Mars Bar)	**Size** (ml or g)	**Price** ($)	**Healthy Choice Symbol** (e.g. heart ♥ or check ✓)	**In Product Binder?** ✓

VENDING AUDIT STEP 3: BRAND NAME FOOD LIST

1. Go to the Brand name Food List www.brandnamefoodlist.ca (register if you have not used the list before)

2. Set up your location/s

3. Set up a score card for each vending machine using your stock lists in step 2

4. Find the products on your stock list for each machine and enter them on your scorecard. If any of the products are not in the BNFL, you will need to get the complete nutritional information from the product label – either from the product in the machine, have someone open the machine or find the product at a grocery store. Submit this information to the Brand Name Food List. When the product gets added to the BNFL you can add it to your scorecard.

5. After ALL of the stock items have been added, submit score cards to National Coordinator. As well, submit a table of the complete nutritional information for each product in ech machine and the % items in each choose/sell category) for the machines audited.

BNFL RESULTS

**Machine #**	Do not sell	Sell Sometimes	Sell Most
**1b**	%	%	%
**2b**	%	%	%
**1s**	%	%	%
**2s**	%	%	%
**1f**	%	%	%
**1r**	%	%	%

Tips for creating your scorecard

- Enter the key word – e.g. snacks or dairy (start your search very general for best success)
- If your machine has more than one of the same product, save time by adding these to the list all at one time using the 'quantity' field

Can't find an item on the list?

- If this is a 'junk food item" likely to be rated as not recommended, enter 'generic' into the keyword field and use one of generic list entries.
- Otherwise submit item to be added to the list (along with the complete nutritional information) to the Brand Name Food List. Once the item has been added to the BNFL you can add it to the scorecard.
