# Supplementary material for: Eat, play, live: a randomized controlled trial within a natural experiment examining the role of nutrition policy and capacity building in improving food environments in recreation and sport facilities
Source: Int J Behav Nutr Phys Act. 2019 Jun 25;16:51. doi: 10.1186/s12966-019-0811-8 (PMC6593504; doi:10.1186/s12966-019-0811-8)
Supplement: Supplementary file 5 — Nutrition Environment Measures Survey–Restaurant reduced item audit. (PDF 311 kb) [file 12966_2019_811_MOESM5_ESM.pdf]

ADDITIONAL FILE 4

Concession Audit

Facility ID: ____________

Date: _________/__________/________ Rater ID: _________
mmm / dd / yyyy (initials)

Number of concessions: _____________

__________ checked by

__________ entered by

_______________ date entered

Concession Description	**Publically** operated and/or funded	**Privately** operated by contractor	**Voluntarily** operated
	☐	☐	☐
	☐	☐	☐
	☐	☐	☐

Concession being audited: (describe): ____

Type of food outlet: ____________________________
(concession or snack bar/café or cafeteria or restaurant)

Concession/ snack bar café has no dedicated seating area. A cafeteria or restaurant has dedicated seating)

A. Hours of operation:

	Monday	Tuesday	Wednesday	Thursday	Friday	Saturday	Sunday
Facility hours:							
Concession hours							

COOKING STYLES (You may need to ask):

a) Does the concession have a deep-fat fryer? No Yes

b) Does the concession have a griddle? No Yes

c) Does the concession have a grill? No Yes

d) Does the concession have an oven? No Yes

MENU REVIEW (You may need to ask questions 1 – 12)
*** Please take photos of all menus. Make sure you can read the menu in the photo! ***

1. Is 1% low-fat, skim, or non-fat milk available? No Yes

2. Is 100% fruit juice available? No Yes
3. Is 100% wheat or whole grain bread available? No Yes
4. Is white or sourdough bread available? No Yes
5. Are ‘regular’ (deep-fat fried) French fries available? No Yes
6. Are baked French fries available? No Yes
7. Are regular (fried) potato/taco etc. chips available? No Yes
8. Are baked chips available (≤ 3 grams fat/serving)? No Yes
9. Is fruit (w/out added sugar) available? No Yes

   # of choices? ___________

10. Are non-fried vegetables without added sauce available? No Yes

    # of choices? ___________

11. Are high fat side-dishes (e.g. snacks, non-entrée, non-packaged foods, purchased separately) available? No Yes

    # of choices? ___________

12. Is low fat or fat-free salad dressing available? No Yes

MAIN DISH/ENTREES and MAIN DISH SALADS (Use primarily menu review)
13. List all **main dishes** (must have significant protein source and at least one other food group. It should be considered a meal. See NEMS rules.)

	1	2	3	4	
Entrées	Whole grain?*	Quality protein?*	Vegetable serving?*	Added fat? ****	Entrée score (1 + 2 + 3 – 4)

* Yes=1, No = 0, CT = CT but score as zero CT = Can't Tell
*** Added fat is from sauce or fried side dish. Yes = minus 1, No = 0, CT = 0

	1	2	3	4	
Entrées	Whole grain?*	Quality protein?*	Vegetable serving?*	Added fat? ****	Entrée score (1 + 2 + 3 – 4)

* Yes=1, No = 0, CT = CT but score as zero CT = Can't Tell
*** Added fat is from sauce or fried side dish. Yes = minus 1, No = 0, CT = 0

	- 1	0	1	2	3
# entrees with score =					

Total # CT in columns 1,2 and 3 = _________________

14. a) Total number of main dishes/ entrees: ___________

    b) # of main dish/entrees with vegetable sides? ___________

    c) # of main dish/entrees with fried sides? ___________

    d) # of main dish/entrees with NO sides? ___________

15. a) Are healthy options available for main dishes and entrees? No Yes

    b) # of choices? ___________ (score = 3 from previous page)

16. a) Total number of main dish salads? ___________

    b) # of healthy main dish salad choices? ___________

17. Are healthy entrees identified on menu? No Yes

18. All-you-can-eat or "unlimited trips"? No Yes

19. Menu notations that encourage large portion sizes. No Yes
    (e.g Super-size items on the menu)

20. Menu notations that discourage special requests. No Yes
    (e.g *No substitutions* or charge for substitutions)
21. Kid's menu? No Yes
    If no kid's menu, skip to question 22

    a. Age limit O 10 & Under O 12 & Under O Other O NA

    b. Any healthy entrees? No Yes

    c. 100% fruit juice No Yes

    d. 1% low-fat, skim or non-fat milk No Yes

    e. Are there any free refills on unhealthy drinks? No Yes

    f. Are there any healthy side items (either assigned or to choose)? No Yes

    g. Can you substitute a healthy side for an assigned unhealthy one? No Yes

    h. Do any entrees that have assigned sides include an assigned healthy side? No Yes

    i. Is an unhealthy dessert automatically included in a kid's meal? No Yes

    j. Are there any healthy desserts(either free or at additional cost)? No Yes

    k. Is nutrition information (e.g. calories or fat) provided on the kid's menu? No Yes

    l. Other unhealthful eating promotion on the kids menu? No Yes

    m. Other healthful eating promotion on the kids menu? No Yes

SIGNAGE AND PROMOTIONS

22. Is nutrition information posted near point-of-purchase, or available in a brochure? No Yes

23. Do signs/table tents/displays <u>encourage healthy</u> eating? No Yes NA

24. Do signs/table tents/displays encourage overeating? No Yes NA
    (all-you-can-eat, super-size, jumbo, grande, supreme, king, size feast descriptors on menu or signage)?

NUTRITION INFORMATION

25. Is nutrition information available on site? No Yes

26. Is nutrition information on the in-concession menu? No Yes

27. Is there nutrition information on the take-away menu? No Yes NA

28. Are healthier menu items identified on the take-away menu? No Yes NA

29. Is nutrition information available on the facility website? No Yes NA

30. Does the facility internet site identify healthier menu items? No Yes NA

31. Is there a venue within the facility that sells alcohol? No Yes

CONCESSION - PACKAGED FOOD AND BEVERAGE AUDIT

Purpose: to audit the packaged foods and beverages available in the concession. Include foods and beverages that would normally be found in a vending machine. Also include fountain drinks and their sizes.

Step 1: Record if the product is at the **P**oint **o**f **P**urchase (POP). POP products are within arm's reach of the cash register and/or directly in your sight line behind the register. (e.g. in a smaller concession there is often limited counter space so products are placed behind the till so customer can see them). Next, record the product details - company name, product's name, flavour, size and price.

Step 2: Find the item in the Product Binder and record y/n. If it is not in the binder, take a good photo of the nutrition label.

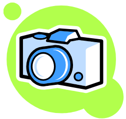

Step 3: Rate the concession packaged products using the Brand Name Food List.

Please **include a photo or two** of the areas where packaged foods/beverages are found in the concession.

**Product #**	**POP?** (y/n)	**Company Name** (e.g. Pepsi, Lays, Doritos etc)	**Product Name & Flavour** (e.g. Gatorade-Berry Blast, Doritos Chips-Salsa, Mars Bar)	**Size** (ml or g)	**Price** ($)	**In Product Binder?** (y/n)
**1**						
**2**						
**3**						
**4**						
**5**						
**6**						
**7**						
**8**						

**Product #**	**POP?** (y/n)	**Company Name** (e.g. Pepsi, Lays, Doritos etc)	**Product Name & Flavour** (e.g. Gatorade-Berry Blast, Doritos Chips-Salsa, Mars Bar)	**Size** (ml or g)	**Price** ($)	**In Product Binder?** (y/n)
**9**						
**10**						
**11**						
**12**						
**13**						
**14**						
**15**						
**16**						
**17**						
**18**						
**19**						
**20**						
**21**						
**22**						

**Product #**	**POP?**<br><br>**(y/n)**	**Company Name**<br><br>(e.g. Pepsi, Lays, Doritos etc)	**Product Name & Flavour**<br><br>(e.g. Gatorade-Berry Blast, Doritos Chips-Salsa, Mars Bar)	**Size**<br><br>(ml or g)	**Price**<br><br>($)	**In Product Binder?** (y/n)
**23**						
**24**						
**25**						
**26**						
**27**						
**28**						
**29**						
**30**						
**31**						
**32**						
**33**						
**34**						
**35**						
**36**						
